# Pupillary responses to invisible brightness escape from attentional modulation

**DOI:** 10.1016/j.isci.2026.115919

**Published:** 2026-04-28

**Authors:** Yung-Hao Yang, Hsin-I Liao

**Affiliations:** 1 Human Information Science Laboratory, Communication Science Laboratories, NTT, Inc., Kanagawa, Japan; 2 Cognitive Informatics Lab, Graduate School of Informatics, Kyoto University, Kyoto, Japan

**Keywords:** biological process, biological sciences, biology of human development, systems biology

## Abstract

The pupillary light response (PLR) is traditionally regarded as a reflex to retinal illumination; however, recent studies have shown that it also reflects perceived or attended brightness. We examined whether pupils respond to perceptually invisible luminance under interocular suppression and most importantly, whether attention modulates such response. In experiment 1, a single bright or dark visual Gabor pattern evoked attenuated but reliable PLRs under interocular suppression, which was independent of feature-based attention manipulation. In experiment 2, spatial attention was directed to one of two Gabors (black and white) using a sustained rapid serial visual presentation task. Spatial attention modulated PLRs when the stimuli were visible but not when they were invisible under interocular suppression. These findings demonstrate that while unconscious brightness signals reach subcortical pathways, spatial attentional modulation of the PLR depends on visual awareness, revealing a cortical-subcortical gating mechanism for pupil control.

## Highlights

• Suppressed brightness evokes reliable but attenuated pupillary light responses
• Spatial attention modulates pupils only when stimuli are consciously perceived
• Awareness acts as a functional gate for spatial attentional control of the pupil

## Graphical abstract

**Figure:**
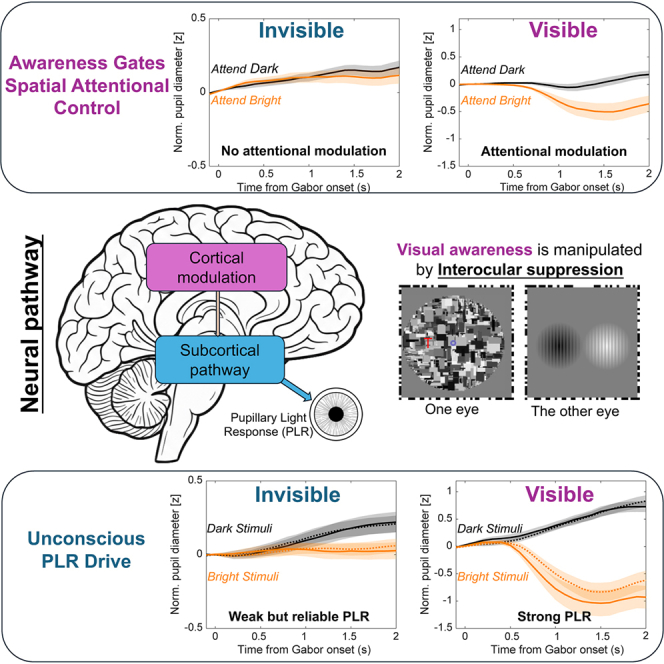

## Introduction

The pupillary light response (PLR) is traditionally regarded as a low-level reflex that regulates retinal illumination through subcortical circuits, including the optic nerves and midbrain. From this traditional perspective, the pupil size merely tracks physical brightness and should be largely independent of higher order processes. However, growing evidence indicates that the PLR is not a purely automatic reflex but is influenced by perceptual interpretation and cognitive factors.^1^ For example, pupils constrict to perceived brightness induced by illusory contours,^2^ mental imagery,^3^ and scene interpretation,^4^^,^^5^ and can also be modulated by attention.^6^^,^^7^^,^^8^^,^^9^^,^^10^^,^^11^ These findings suggest that the PLR provides a physiological window into the interplay between sensory input and top-down processing.

A central question is whether the PLR depends on visual awareness. Early binocular rivalry studies reported attenuated pupil responses when a luminance stimulus is perceptually suppressed, suggesting that the PLR reflects perceived brightness rather than retinal input alone.^12^^,^^13^^,^^14^^,^^15^ More recently, pupillometry has been used as an objective measure of perceptual dominance during rivalry, showing that pupil dynamics reflect perceptual state and change gradually around perceptual transitions.^16^ In contrast, Sperandio and colleagues^17^ reported that PLR to bright scenes is abolished when the scenes are out of awareness through continuous flash suppression (CFS),^18^ a dichoptic masking paradigm that induces interocular suppression. However, other studies indicate that pupil responses can persist without awareness of the visual inputs. For example, reliable pupillary responses were found in response to suppressed luminance or chromatic changes,^19^ familiar faces,^20^ affective images,^21^ social threat,^22^ and threat-conditioned stimuli.^23^ Together, these findings suggest that suppression may reduce the effective strength of luminance signals driving the pupil, rather than eliminating pupillary responses entirely. Consistent with this idea, early clinic work on suppression in strabismus linked changes in eye dominance under unequal luminance input to corresponding pupillary responses,^24^ and pupil perimetry studies reported attenuated response amplitudes in localized cortical visual field deficits.^25^ These observations suggest that early brightness information may reach subcortical pupillary pathways independently of awareness, though the conditions under which awareness or attention modulate such responses requires further explorations.

Attention provides an important top-down route through which perception can influence the PLR. When observers covertly attend to a bright rather than a dark region while keeping their fixations steady, the pupil constricts more strongly despite total luminance being constant.^7^ A subsequent study^9^ extended this finding to feature-based attention, showing that when bright and dark dot surfaces overlapped in space, attending to the bright surface resulted in smaller pupil sizes than attending to the dark one. Beyond luminance selection, pupil dynamics can also track moment to moment attentional allocation in demanding visual tasks,^6^ supporting the broader view that pupillometry provides an integrated readout of distinct attentional networks.^26^ These results suggest that attention can modulate pupil size through cortical-brainstem coupling.

Whether attentional modulation of the PLR depends on visual awareness, however, remains unresolved. Prior work on unconscious processing shows mixed findings: some reported that feature-based attention enhances the processing of suppressed gratings,^27^ and spatial attention facilitates adaptation to suppressed stimuli ranging from low-level patterns^28^ to high-level facial expressions.^29^ In contrast, others found no evidence of attention modulation^30^ or even opposite patterns, where unattended but semantically meaningful stimuli survived suppression more effectively than attended counterparts.^31^ These discrepancies suggest that attentional influences on unconscious perception are context-dependent, possibly varying with task demands and stimulus complexity. Importantly, these studies address unconscious perceptual processing rather than pupillary responses, leaving open the question of whether attention can influence the PLR when stimuli are rendered invisible.

Here, we consider feature-based and spatial attention, along with interocular suppression, to examine how awareness and attention jointly shape the PLR. In both experiments, we focused on the CFS condition to test pupillary responses to suppressed brightness while including a small number of visible trials as a reference to verify normal pupillary responses and task compliance. In experiment 1, we presented a single bright or dark Gabor and asked whether suppressed brightness evokes a reliable PLR, and most importantly, whether feature-based attention to luminance (vs. orientation) modulates this response. In experiment 2, we presented a bright and a dark Gabor simultaneously, such that their opposing luminance signals should largely cancel out with each other at the retinal level and tested whether spatial attention to one location can bias pupil size, and whether this attentional modulation is preserved under suppression. Together, these experiments test when luminance-driven PLRs persist under suppression and whether attentional modulation requires awareness.

## Results

### Experiment 1: Feature-based attention

**Figure 1:**
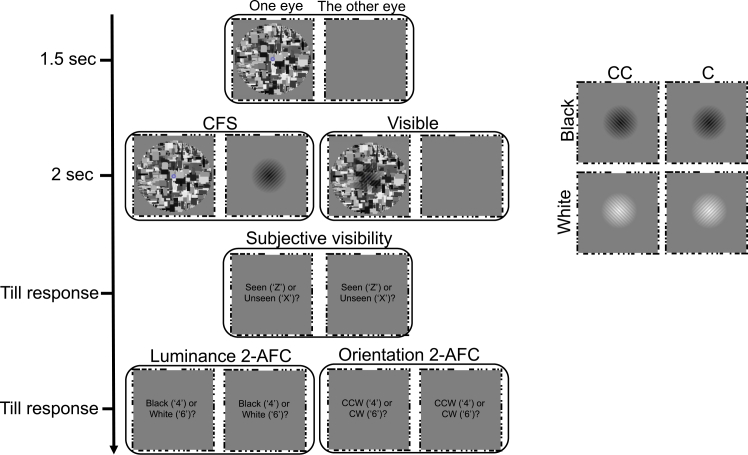
The procedure of experiment 1 (feature-based attention) Stimuli were presented dichoptically, with a dynamic Mondrian mask shown to one eye and a Gabor patch shown to the other eye (continuous flash suppression, CFS condition) or superimposed in the same eye (visible condition). Each trial began with a 1.5 s baseline in which only the dynamic mask was presented, followed by 2 s of Gabor presentation. The Gabor stimuli consisted of four combinations of luminance and orientation: black-clockwise (CW), white-clockwise, black-counterclockwise (CCW), and white-counterclockwise. After stimulus offset, participants first reported subjective visibility (“seen” by pressing “Z” key; “unseen” by pressing “X” key). They then performed a two-alternative forced-choice (2-AFC) discrimination. In the luminance task, participants judged (or forced to guess) whether the Gabor was black (by pressing “4” on the number pad) or white (by pressing “6” on the number pad). In the orientation task, they judged whether the Gabor was tilted counterclockwise (“4”) or clockwise (“6”).

Participants viewed bright or dark Gabor patches under CFS or visible conditions while attending either to the patch’s luminance or orientation (Figure 1). Trials in which participants reported “seen” in the CFS condition (1.60%) or “unseen” in the visible condition (1.91%) were excluded. Objective awareness was assessed at the individual level using the chi-squared procedure. One participant showed above-chance discrimination (accuracy = 0.641, *p* < 0.001) and was excluded. The remaining group performed at chance (mean = 0.499, SD = 0.030), and trial-wise mixed-effects logistic regression confirmed no deviation from chance (estimated accuracy = 0.499, z = −0.18, *p* = 0.855), supporting effective suppression.

#### Pupil responses to luminance, visibility, and feature-based attention

**Figure 2:**
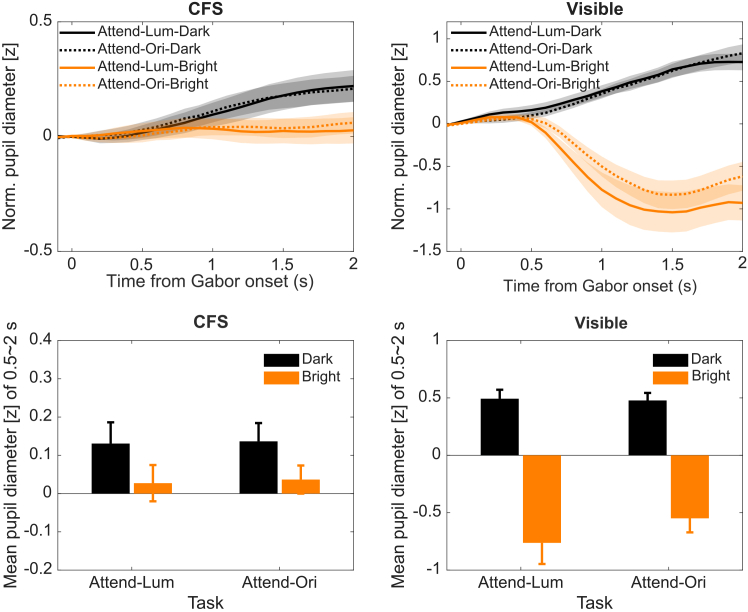
Pupillary light responses in experiment 1 (feature-based attention) (Top) Normalized pupil diameter change (*Z* scored) is shown as a function of time from Gabor onset under continuous flash suppression (CFS; left) and visible conditions (right). Trial-averaged pupil traces are parameterized when responding to dark (black) and bright (orange) Gabors under two attention tasks: attend-luminance (solid lines) and attend-orientation (dotted lines). Shaded areas represent ±1 SEM across participants. (Bottom) Average pupil size during the 0.5–2.0 s post-stimulus window for each condition. Error bars represent ±1 SEM.

Figure 2 shows pupillary light responses across feature-based attention and awareness conditions. First, both frequentist and Bayesian three-way repeated-measures ANOVAs were conducted on mean pupil diameters (0.5–2 s after Gabor onset), with factors of visibility (CFS vs. visible), Luminance (dark vs. bright), and attention task (attend-luminance vs. attend-orientation).

The analysis revealed a significant main effect of luminance, *F*(1,16) = 44.68, *p* < 0.001, *BF*_incl_ = 3911.99 (Bayes factor comparing models that contain the effect to equivalent models stripped of the effect), indicating larger pupil size in response to dark Gabors than bright Gabors. There was also a significant visibility × luminance interaction, *F*(1,16) = 45.18, *p* < 0.001, *BF*_incl_ = 4132.71, reflecting that the luminance-driven PLR was robust in the visible condition but attenuated under CFS. Simple effects analyses showed that dark Gabors evoked larger pupil size than bright Gabors in all combinations of the awareness and attention conditions: *F*(1,16) = 14.98, *p* = 0.001, *BF*_10_ = 31.24 in attend-luminance task and *F*(1,16) = 14.95, *p* = 0.001, *BF*_10_ = 26.64 in attend-orientation task under CFS; *F*(1,16) = 36.13, *p* < 0.001, *BF*_10_ = 1296.97 in attend-luminance task, and *F*(1,16) = 42.77, *p* < 0.001, *BF*_10_ = 3126.17 in attend-orientation task when the Gabor was visible. No significant main effect of attention was observed, *F*(1,16) = 1.53, *p* = 0.234, *BF*_incl_ = 0.45, nor were there significant interactions involving attention (all ps > 0.129, *BF*s < 1.17), suggesting that feature-based attention did not modulate the pupillary response.

To test the robustness of these findings, as the trial number was uneven between the CFS and visible conditions and to control for potential oculomotor confounds such as the pupil foreshortening error,^32^ we analyzed the trial-level data using a linear mixed-effects (LME) model. We included both horizontal and vertical gaze positions in the model to examine whether the LME visibility × luminance interaction remained when the gaze position was controlled. Likelihood ratio tests (LRT) showed that the inclusion of these gaze covariates significantly improved model fit compared to a reduced model excluding them (*χ*^2^(2) = 12.30, *p* = 0.002). Both horizontal (*t* = −3.31, *p* < 0.001) and vertical (*t* = 2.40, *p* = 0.016) gaze positions showed significant influences. We then included both gaze positions in the full model for the formal analysis. Results showed the significant effects of visibility, *F*(1, 743.9) = 19.60, *p* < 0.001, luminance, *F*(1, 1954.9) = 440.26, *p* < 0.001, and the visibility × luminance interaction *F*(1, 1954.7) = 311.13, *p* < 0.001. Furthermore, the luminance effect persisted under CFS (*β* = 0.12, *t* = 3.61, *p* < 0.001) and the visible condition (*β* = 1.39, *t* = 21.79, *p* < 0.001), indicating the attenuation of the luminance-driven PLR under suppression persists even after controlling for trial-by-trial gaze fluctuations.

With this increased sensitivity, the LME model detected a significant visibility × attention interaction, *F*(1, 1956.0) = 8.10, *p* = 0.004. The average pupil size was larger in the CFS than in the visible conditions. This could be explained by increased cognitive load, as the participants might have found the tasks more difficult under conditions of limited visibility. Post hoc analysis indicated that under the visible condition, pupil size was larger in the attend-orientation than attend-luminance task (*β* = −0.13, *t* = −2.01, *p* = 0.044), whereas under the CFS condition, the pattern was reversed (*β* = 0.08, *t* = 2.31, *p* = 0.021). A parsimonious explanation for the former could lie in the higher cognitive load required to discriminate orientation when the stimulus was visible. Nevertheless, the three-way interaction remained non-significant (visibility × luminance × attention, *p* = 0.254). Taken together, these results suggest that while attention may subtly shift baseline pupil diameter, it does not modulate the strength of the PLR itself.

#### Bayesian evidence for the absence of unconscious feature-based attentional modulation

To quantify the evidence against attentional modulation under suppression, we performed a Bayesian model comparison on the CFS data. We tested a LME model including the luminance × attention interaction against a null model containing only the main effects. The comparison favored the null model (Δ*BIC* = 6.71), yielding a Bayes factor *BF*_01_ = 28.64. This indicates strong evidence against the hypothesis that feature-based attention interacts with luminance processing to modulate the PLR under suppression.

In sum, experiment 1 provided two key insights. First, it showed that interocular suppression attenuates but does not abolish the luminance-driven PLR, establishing a quantifiable baseline for unconscious processing. Second, and critical to our research question, feature-based attention failed to modulate this preserved response. This null result suggests that feature-based attention is insufficient to penetrate suppression, or that modulation of the PLR requires stronger competition between concurrently available visual representations. Consequently, this motivated experiment 2, where we introduced a spatial attention task to determine if spatially focusing resources on a specific location, rather than a feature dimension, is able to modulate the unconscious PLR.

### Experiment 2: Spatial attention

**Figure 3:**
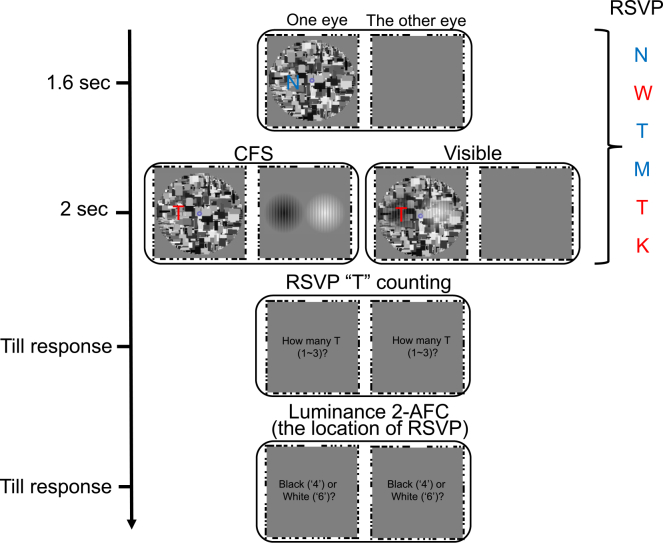
The procedure of experiment 2 (spatial attention) Stimuli were presented dichoptically, with a dynamic Mondrian mask shown to one eye and a pair of bright and dark Gabors shown to the other eye (CFS condition) or superimposed in the same eye (visible condition). Each trial began with a 1.6 s baseline in which only the dynamic mask and an RSVP stream of letters were presented, followed by 2 s of Gabor presentation. The RSVP stream (red or blue letters) was always presented at the center of one Gabor, with the intention of directing spatial attention to either the bright or dark Gabor. After stimulus offset, participants performed two tasks. First, they reported the number of target “T” letters (regardless of colors) in the RSVP sequence (number from 1 to 3). Second, they judged (or forced to guess) the luminance of the Gabor superimposed at the RSVP location (if black, pressing “4,” if white, pressing “6” on the number pad). Inaccurate RSVP trials were excluded from analysis.

In the second experiment, participants viewed a pair of bright and dark Gabors while performing a rapid serial visual presentation (RSVP) task at one of the locations, thereby directing spatial attention to either the bright or dark Gabor (Figure 3). Five participants showed above-chance objective discrimination and were excluded (mean = 0.726, SD = 0.063). In the remaining sample (*N* = 12), discrimination performance was at chance (mean = 0.520, SD = 0.026), and a mixed-effects logistic regression did not reveal a reliable deviation from chance (estimated accuracy = 0.521, z = 1.73, *p* = 0.084), indicating that suppression was effective. Accuracy of the RSVP task was similarly high in both CFS and visible conditions (92.10% vs. 92.28%), indicating that attentional engagement with the RSVP stream was comparable across the two visibility conditions.

#### Pupil responses to visibility and spatial attention

**Figure 4:**
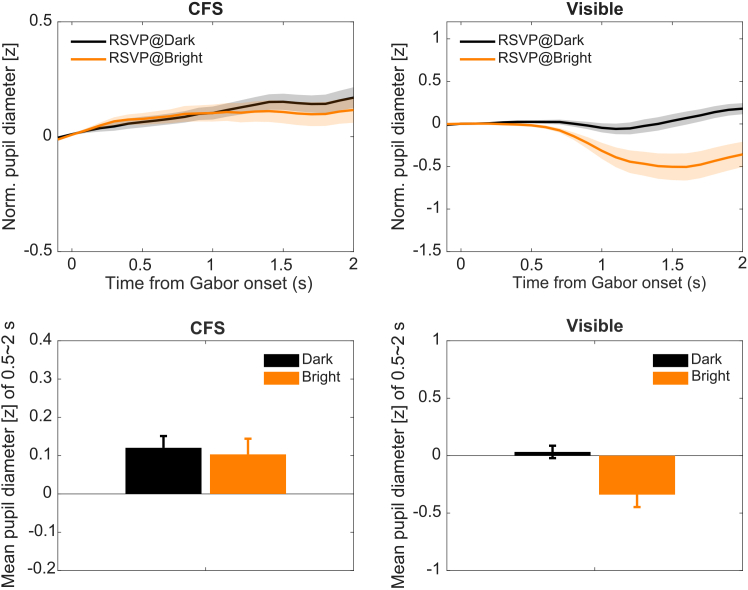
Pupillary light responses in experiment 2 (spatial attention) (Top) Normalized pupil diameter change (*Z* scored) is plotted as a function of time from Gabor onset under continuous flash suppression (CFS; left) and visible conditions (right). Trial-averaged pupil traces are parameterized when the RSVP stream was superimposed on the dark (black) and bright (orange) Gabors, (RSVP@dark vs. RSVP@bright). Shaded areas represent ±1 SEM across participants. (Bottom) Average pupil size during the 0.5–2.0 s post-stimulus window. Error bars represent ±1 SEM.

Figure 4 illustrates pupillary light responses across conditions of spatial attention and awareness. Both frequentist and Bayesian two-way repeated-measures ANOVAs were conducted on mean pupil responses (0.5–2 s after stimulus onset), with factors of visibility (CFS vs. visible) and attended-luminance at the RSVP location (dark vs. bright). The results showed significant main effects of visibility, *F*(1,11) = 12.29, *p* = 0.005, *BF*_incl_ = 10.60, and attended-luminance, *F*(1,11) = 12.70, *p* = 0.004, *BF*_incl_ = 3.53. There was also a significant visibility × attended-luminance interaction, *F*(1,11) = 8.87, *p* = 0.013, *BF*_incl_ = 36.78. Simple effects analyses showed that attended luminance had no effect in the invisible (CFS) condition, *F*(1,11) = 0.32, *p* = 0.585, *BF*_10_ = 0.35. By contrast, in the visible condition, attended luminance significantly modulated pupil responses, *F*(1,11) = 11.15, *p* = 0.007, *BF*_10_ = 9.14, with larger pupil size when attending to the dark Gabor than when attending to the bright Gabor. Thus, spatial attention influenced pupil dynamics only when the Gabors were consciously perceived, and not when they were suppressed from awareness.

To rigorously test this dissociation while accounting for fixation stability, we analyzed the trial-level data using a LME model including gaze positions, following the same procedure as in experiment 1. This model also explicitly controlled for horizontal (*t* = −4.01, *p* < 0.001) and vertical (*t* = −0.64, *p* = 0.526) gaze positions, ensuring that results were not driven by the pupil foreshortening error. LRT indicated that including gaze covariates significantly improved the model fit compared to a reduced model (*χ*^2^(2) = 16.5, *p* < 0.001).

The results showed significant main effects of visibility (*F*(1, 1951.8) = 158.07, *p* < 0.001) and attended-luminance (*F*(1, 1952.4) = 81.88, *p* < 0.001). Crucially, the model supported a robust visibility × attended-luminance interaction (*F*(1, 1952.4) = 61.14, *p* < 0.001). Post hoc contrasts on the LME model showed that in the visible condition, attending to the dark Gabor resulted in significantly larger pupil sizes than attending to the bright Gabor (*β* = 0.46, *t* = 9.98, *p* < 0.001). However, in the CFS condition, this modulation was statistically absent (*β* = 0.03, *t* = 1.15, *p* = 0.251), demonstrating that spatial attention to luminance does not elicit a PLR when the stimulus is suppressed.

#### Bayesian evidence for the absence of unconscious spatial attentional modulation

Similarly, we assessed the evidence for spatial attentional modulation under suppression. We compared a model that included the attended-luminance factor against a null model that excluded it using data from the CFS condition. The comparison favored the null model (Δ*BIC* = 5.79), yielding a Bayes factor *BF*_01_ = 18.13, providing strong evidence in favor of the null hypothesis (i.e., that spatial attention does not modulate the pupil under suppression).

#### Control for oculomotor confounds: Fixation instability cannot explain the attention-modulated pupil responses

Despite of controlling the effect of gaze positions in the LME model, we concerned whether the participant maintained their fixation steadily across different conditions, and most importantly, whether the physical retinal illuminance inputs associated with the gaze position explains the attention-modulated pupil responses. We first inspected the horizontal gaze position distribution. Although most of the gaze positions clustered around the central position, there was noticeable bias toward the task-relevant RSVP streams (Figure S1). We therefore performed a correlation analysis between trial-by-trial pupil size with the Gaze-to-stimulus distance (defined as the absolute horizontal deviation from the center of the attended Gabor patch: |*Gaze*_*x*_-*Target Center*|). This analysis aimed to dissociate top-down attentional effects from the physical “foveal light reflex.” Under visible condition, the pupil size correlated with gaze position when attending to dark Gabor (*r* = −0.34, *p* < 0.001) but not when attending to bright Gabor (*r* = −0.05, *p* = 0.363). Under CFS condition, the regression lines between the gaze position and pupil size showed similar trend regardless of whether attending to dark or bright Gabors (*r* = −0.07 and −0.09, respectively), indicating that pupil size could not be explained by the physical Gabor patch’s luminance (Figure S2). The overall results suggested that while the attention-modulated PLR observed in the visible condition may be partly explained by eye movement (e.g., participants looking closer to the target when attending to the dark Gabor), this does not hamper the conclusion of the absence of attention modulation under the CFS condition.

## Discussion

The present study aimed to clarify two questions about the pupillary light response (PLR): whether pupils respond to suppressed brightness, and whether such responses are modulated by attention. We found that even when the stimuli were suppressed from awareness under CFS, they still elicited reliable PLRs, with pupils dilated to dark Gabors and constricted to bright Gabors. This indicates that unconscious brightness signals can reach subcortical pupillary pathways. However, attentional modulation of the PLR showed a clear dissociation: feature-based attention produced no measurable effect in either visibility condition, whereas spatial attention modulated the PLR only when the stimuli were consciously visible.

### Feature-based attention and the PLR

In experiment 1, bright and dark Gabors produced robust PLRs even when the stimuli were suppressed, indicating an awareness-independent component of the pupil responses. However, directing attention to the luminance versus orientation did not alter such pupillary responses. This null result contrasts with Binda et al. (2014),^9^ who reported that attending to bright versus dark overlapping dot surfaces led to smaller versus larger pupils. However, it aligns with a recent failure to replicate feature-based pupillary modulation in a Navon task,^33^ suggesting that feature-based attention may not universally drive the PLR.

While the strong suppression in the CFS condition likely dampened feature signals, the absence of modulation in the visible condition points to a more fundamental constraint, the context of the stimuli. In Binda’s paradigm, two concurrent, perceptually segregated surfaces competed for selection, providing a strong cue for feature-based gain modulation. In our design, a single tilted Gabor containing both luminance polarity and orientation was presented per trial, and observers were instructed to attend to one feature dimension. Without competition between concurrent surfaces or locations, feature-based selection of brightness representations may have been too weak to influence subcortical PLR pathways. These findings suggest that feature-based attention modulates the PLR primarily when multiple representations coexist and compete for selection, rather than during selective attention to a single, non-competing object.

### Spatial attention, awareness, and the PLR

In experiment 2, we introduced spatial competition by presenting both bright and dark Gabors simultaneously. When the stimuli were consciously visible, directing attention to one location clearly modulated pupil size^7^^,^^8^; attending to the dark Gabor increased pupil dilation, whereas attending to the bright Gabor enhanced constriction. When the same displays were rendered invisible under CFS, this modulation disappeared. Pupils simply reflect the net luminance of the display, consistent with cancellation between opposing brightness influences. Notably, because the RSVP task was intentionally designed to occupy a low attentional load,^34^ the null effect under suppression cannot be attributed to excessive attentional engagement. Rather, it indicates that awareness is required for spatial attention to influence the PLR.

Our findings reveal a clear dissociation: spatial attention modulated the PLR when stimuli were visible, but this modulation disappeared under interocular suppression. This result contributes to the ongoing debate regarding the limits of top-down selection. Previous literature has yielded conflicting conclusions. One view suggests that spatial attention and awareness are fully dissociable, supported by findings that spatial attention can enhance the processing of invisible stimuli, such as strengthening adaptation aftereffects^28^ and modulating the blood-oxygen-level-dependent (BOLD) signal.^35^ In contrast, other influential work argues for a stricter dependency. For instance, Kanai et al.^27^ demonstrated that spatial attention facilitates orientation processing only when the inducing stimulus is visible, implying that top-down spatial selection cannot target neural representations that are insulated from awareness. Our pupillometric data align with this latter view.^27^ Despite using a paradigm, where spatial attention clearly biased the pupil in the visible conditions, the subcortical PLR pathway remained functionally insulated from these top-down signals during suppression. This suggests that while attention may modulate certain cortical representations without awareness,^28^ the modulation of subcortical reflexes like the PLR appears strictly contingent upon the conscious availability of the target.

### Neural mechanisms of the dissociation

This dissociation can be explained by the hierarchical organization of pupillary control. The prefrontal cortex is known to exert top-down control over brainstem pupillary centers.^36^ A recent framework proposes that pupillometry serves as an integrated readout of distinct attentional networks, specifically distinguishing between the global arousal system (mediated by the locus coeruleus, LC) and the spatial orienting system (mediated by the superior colliculus, SC).^26^ The LC-norepinephrine system tracks task utility and cognitive effort, which likely explains the average pupil size differences we observed in experiment 1 (e.g., increased load in the CFS than the visible condition). In contrast, the modulation of the PLR itself is thought to rely on the SC, which integrates retinal inputs with top-down spatial priority signals from the frontal eye fields (FEFs).^26^ Our findings imply a functional dissociation between these hubs: while the subcortical reflex pathway (retina to pretectum) remains active under suppression, the top-down projection (cortex to SC to pupil) appears functionally gated by awareness. Without the reentrant cortical processing associated with conscious perception, the SC may fail to receive the top-down “priority map” necessary to bias the pupillary light reflex, leaving the system insulated from attentional modulation.

### Conclusion

Together, these results show that the PLR comprises two interacting components: an awareness-independent, subcortical brightness response and an awareness-dependent, cortical attentional modulation. On one hand, our results align with studies showing that unconscious visual signals can still drive subcortical pupillary pathways.^19^^,^^20^^,^^21^^,^^22^^,^^23^ On the other hand, the absence of attentional modulation under suppression highlights a boundary condition that top-down attentional influences on the PLR require conscious perception.

These findings support the view that while the subcortical reflex machinery is robust to suppression, the functional connectivity required for top-down control by awareness. Thus, pupillometry serves as a powerful noninvasive tool for dissociating the automatic, awareness-independent components of vision from the controlled awareness-dependent mechanisms of attention.

### Limitations of the study

We acknowledge three limitations. First, we restricted our investigation to feature-based and spatial attention. It remains possible that other forms of attention (e.g., temporal expectation and object-based attention) might interact differently with pupillary dynamics under suppression. Second, we employed an RSVP task to guarantee robust, sustained spatial attention. The design inherently directed attention toward the dominant eye (the mask), which may have “locked” attentional resources. Future studies utilizing endogenous cues could determine whether reducing mask engagement facilitates modulation. Finally, the use of active shutter glasses for dichoptic presentation resulted in a data loss rate of approximately 31%–40%. These glasses attenuate the eye-tracker’s infrared signal (analogous to wearing sunglasses) and reduce pupil contrast. Although our LME analysis explicitly controlled for gaze position despite these gaps, future work utilizing optimized tracking setups would further validate the stability of these findings.

## Resource availability

### Lead contact

Further information and requests for resources and reagents should be directed to and will be fulfilled by the lead contact, Hsin-I Liao (hsini.liao@ntt.com).

### Materials availability

This study did not generate new unique reagents.

### Data and code availability

• Raw data have been deposited on the Open Science Framework and are publicly available (DOI: https://doi.org/10.17605/OSF.IO/CGVW8).
• The analysis codes have been deposited on the Open Science Framework and are publicly available (DOI: https://doi.org/10.17605/OSF.IO/CGVW8).
• Any additional information required to reanalyze the data reported in this paper is available from the lead contact upon request.

## Acknowledgments

We thank Dr. Marnix Naber and anonymous reviewers for their thoughtful reviews. Their insights contributed greatly to the refinement of this manuscript.

## Author contributions

Conceptualization, methodology, investigation, writing – original draft, writing – review and editing, and resources, Y.-H.Y. and H.-I.L.

## Declaration of interests

The authors declare no competing interests.

## Declaration of generative AI and AI-assisted technologies in the writing process

During the preparation of this work, the authors used Grammarly in order to check grammar. After using this tool or service, the authors reviewed and edited the content as needed and takes full responsibility for the content of the publication.

## STAR★Methods

### Key resources table

**Table:** 

REAGENT or RESOURCE	SOURCE	IDENTIFIER
**Deposited data**		

Raw data and analysis codes	OSF storage	https://doi.org/10.17605/OSF.IO/CGVW8

**Software and algorithms**		

MATLAB 2018b	https://jp.mathworks.com/products/matlab.html	RRID:SCR_001622
Psychophysics Toolbox Version 3	http://psychtoolbox.org/	RRID:SCR_002881
R package: lmerTest	http://cran.r-project.org/package/lmerTest	RRID: SCR_015656
R package: lme4	https://cran.r-project.org/web/packages/lme4	RRID:SCR_015654
JASP (Jeffrey’s Amazing Statistics Program)	https://jasp-stats.org	RRID:SCR_015823
G∗Power	http://www.gpower.hhu.de/	RRID:SCR_013726

**Other**		

SR Research EyeLink Eye Trackers (Eyelink 1000 Desktop Mount)	http://www.sr-research.com	RRID:SCR_009602
NVIDIA 3D Vision 2	https://www.nvidia.com/	N/A

### Experimental model and study participant details

Eighteen observers participated (12 females, 6 males; *M*_*age*_ = 33.06, *SD*_*age*_ = 10.44) in Experiment 1 and seventeen (12 females, 5males; *M*_*age*_ = 36.24, *SD*_*age*_ = 10.44) in Experiment 2. All were of Japanese ancestry and had normal or corrected-to-normal vision. The study was approved by the Ethics Committee of Nippon Telegraph and Telephone Corporation, Japan (H30-011). Written informed consent was obtained from all participants prior to participation. Sample sizes were determined based on established precedents in attention and pupillometry research,^8^^,^^37^ which demonstrate robust effects with N between 15 and 20. Following exclusions for above-chance objective awareness, the final analyzed samples consisted of N = 17 for Experiment 1 and N = 12 for Experiment 2. Sensitivity power analyses (G∗Power 3.1) supported that these final samples provided sufficient statistical power (*α* = .05, power = .80, *r* = .50) to detect medium-to-large effects. Specifically, Experiment 1 (8 measurements) was sensitive to *f* = 0.24 ($\eta_{p}^{2}\approx .05$), and Experiment 2 (4 measurements) was sensitive to *f* = 0.36 ($\eta_{p}^{2}\approx .11$). Given that the pupillary light reflex is a fundamental physiological response, sex and gender were not expected to influence the results.

### Method details

#### Apparatus

All stimuli and experimental procedures were programmed in MATLAB 2018b using the Psychophysics Toolbox Version 3. Dichoptic stimulus presentation was achieved using NVIDIA 3D Vision 2 active shutter glasses paired with a 120-Hz monitor (Asus VG248QE, 24-inch, 1920 × 1080 resolution) at a viewing distance of 57cm. The monitor was set to standard sRGB mode with a nominal gamma of 2.2. Pupil diameter was recorded monocularly at a 1000-Hz sampling rate with the Eyelink 1000 Desktop Mount system.

#### Stimuli

To facilitate binocular fusion, each eye viewed a 30 ° × 30 ° outer frame composed of alternating black-and-white rectangles on a mid-gray background ([128 128 128] RGB), which remained visible throughout the experiment. Two types of visual stimuli were used**.** The first was an interocular mask**,** a Mondrian-style grayscale circle (diameter: 28°) composed of 5,000 overlapping square patches (0.02°–1.10°) of random grayscale luminance. The grayscale values were randomized uniformly between black (0.3 cd/m^2^) and white (350 cd/m^2^), yielding 100% Michelson contrast. The pattern was refreshed at 10Hz to maintain continuous suppression. The second was Gabor patches (diameter: 13° viewing angle)**,** which served as the critical stimuli for eliciting pupillary responses. The Gabors were defined by a sinusoidal grating (2.2 cycles/degree) enclosed within a Gaussian envelope (*σ*= 2.8°). To generate the “Bright” and “Dark” conditions, the pixel intensity of the gratings was modulated relative to the mid-gray background (RGB 128; ≈76 cd/m^2^). The Bright Gabor (RGB 192–255) had a nominal mean luminance of 260 cd/m^2^, while the Dark Gabor (RGB 0–64) had a nominal mean luminance of 4 cd/m^2^.

In Experiment 1, a single Gabor patch (diameter: 13° viewing angle) was presented in the center of the outer frame. Its luminance (black or white) and orientation (±45°, clockwise [CW] or counterclockwise [CCW]) were varied.

In Experiment 2, a pair of Gabor patches (diameter: 13°; orientation: 0°, one black and one white) was presented side by side with a center-to-center distance of 14°. To direct spatial attention, a rapid serial visual presentation (RSVP) stream of letters (['T', 'N', 'Z', 'M', 'L', 'K', 'W']; 4.5° in size) appeared in red ([255 0 0]) or blue ([0 0 255]) at the center of one Gabor.

In both experiments, a large circular mask covered most of the visual field. To manipulate visual awareness of the Gabor patches, they were presented either to the eye opposite the mask (CFS condition) or to the same eye (Visible condition). Under continuous flash suppression (CFS), the Gabor shown to the non-dominant eye was suppressed by the mask in the dominant eye, rendering it subjectively invisible (i.e., suppressed from awareness) for a period of time, despite its physical suprathreshold contrast. In the Visible condition, the Gabor was superimposed on the mask and presented to the same eye.

#### Design

Experiment 1 investigated feature-based attention using a within-subject design with three factors: visibility (CFS or visible), Gabor luminance (black or white) and attended feature (luminance or orientation). Four stimulus types were included: black-CW, white-CW, black-CCW, and white-CCW. To manipulate feature-based attention, participants judged either whether the Gabor was black or white (attend-luminance) or whether it was tilted clockwise or counterclockwise (attend-orientation). The experiment consisted of four blocks: two under attend-luminance instruction and two under attend-orientation instruction, with block order counterbalanced across observers. Each block consisted of 88 trials, including 80 CFS trials and 8 visible trials, with equal representation of the four stimulus types. All the trials were presented in random order within each block.

Experiment 2 investigated spatial attention using a within-subject design with two factor: visibility (CFS or visible) and attended luminance (black or white). Spatial attention was manipulated by assigning the RSVP task to the location of either the black or the white Gabor. The experiment included two blocks of 112 trials each, consisting of 80 CFS trials and 32 visible trials. The left-right arrangement of the black and white Gabor pair and the RSVP task location (left vs. right) were counterbalanced across trials.

In both experiments, stimuli were presented under two visibility conditions: CFS (Gabor suppressed by a dynamic mask in the other eye) and Visible (Gabor shown to the same eye as the mask). Although visibility (CFS vs. Visible) was included as a factor in the statistical analysis, it was not part of the planned attentional design. The two visibility conditions were intentionally unbalanced in trial number because visible trials served primarily as sanity-check trials to verify normal PLR responses and task compliance. Thus, visibility should be interpreted as an additional analytical factor, not a fully crossed design variable.

#### Procedure

At the beginning of each block, participants aligned the outer frames using arrow keys to achieve stable binocular fusion. In Experiment 1, they were instructed before each block to attend either to the luminance or to the orientation of the Gabor. Each trial began with a 1.5 s baseline period during which only the dynamic mask was presented to one eye to habituate pupil responses. A Gabor patch was presented for 2 s. After stimulus presentation, participants first judged subjective visibility by answering the question “Did you see the Gabor?” with the “z” (seen) or “x” (unseen) keys, and then performed an objective discrimination task: in the attend-luminance condition, indicating whether the Gabor was black or white; and in the attend-orientation condition, whether it was tilted clockwise or counterclockwise.

In Experiment 2, the dynamic mask and RSVP stream were presented during the first 1.6 s baseline, serving both to habituate pupillary responses and to provide a spatial cue for attention. This baseline duration was extended slightly relative to Experiment 1 (1.5 s) to accommodate the temporal structure of the RSVP stream (six items × 600 ms) and ensure precise synchronization with the subsequent 2 s Gabors (one black and one white) presentation (1.6 + 2 = 0.6∗6). The RSVP stream was always superimposed on the dynamic mask in the dominant eye, spanned the full trial duration (3.6 s). It consisted of six letters presented sequentially for 300 ms each, with a 300-ms inter-stimulus interval. The target was a red or blue “T,” which appeared randomly one to three times per trial. We adopted this ‘low load’ condition (detecting T regardless of its color) following Bahrami and colleagues^21^ to maintain sustained attention without excessive engagement. The remaining letters were randomly selected from ['N', 'Z', 'M', 'L', 'K', 'W'] to serve as distractors, with the constraint that no letter was repeated consecutively. Their color (red or blue) was randomly assigned on each presentation. Participants were instructed to count how many target ‘T’ letters appeared in the RSVP stream (1, 2, or 3). After each trial, participants reported (1) the number of “T” by pressing the corresponding numeric key (‘1’, ‘2’, or ‘3’), and (2) the luminance of the Gabor located at the RSVP position by pressing “4” for black or “6” for white. Given the high response load of this dual-task design, a separate subjective visibility rating was not included; awareness of the Gabor patches was instead rigorously quantified via the objective luminance discrimination performance.

### Quantification and statistical analysis

#### Pupil data processing

##### Calibration

Before each block, a five-point calibration and validation procedure was performed to ensure that pupillary responses could be reliably recorded through the shutter glasses.

##### Preprocessing

Pupil data were processed with custom MATLAB scripts at a sampling rate of 1000 Hz. Blink events were identified from the Eyelink event logs by detecting “EBLINK” markers. To reduce blink-related artifacts, pupil and gaze samples from 150 ms before to 150 ms after each blink were removed. Pupil diameter traces were then smoothed using a custom routine. This function treated zeros in the raw signal (indicating blinks or missing samples) as missing data, interpolated across these gaps using piecewise cubic Hermite interpolation (PCHIP), and produced a continuous, blink-corrected signal suitable for trial-wise analysis. For each trial, data quality was quantified by calculating the proportion of missing gaze samples in the 0–2 s window after stimulus onset. This trial-level quality index was 39.41% in Experiment 1 and 31.32% in Experiment 2.

##### Data exclusion

In Experiment 1, CFS trials in which the participant reported “seen” and visible trials in which the participant reported “unseen” were excluded based on subjective visibility reports. In Experiment 2, trials in which participants failed to correctly report the count of target 'T's in the RSVP task were defined as attentional lapses and were excluded from further analysis. In both experiments, to confirm that the Gabor stimuli remained invisible at the participant level, we applied an objective performance check. Specifically, we tested discrimination accuracy in the CFS condition with a chi-square analysis. Participants with p > .05 in this analysis were retained for averaging, while those failing the check were excluded for further analysis.

##### Trial-wise normalization

For each trial, pupil signals were aligned to Gabor onset and segmented from −1 s to +2 s relative to this time. Pupil size was z-scored within each subject across the full recording to stabilize variance across trials and then baseline-corrected by subtracting the mean over the −100 to 0 ms pre-stimulus interval.

#### Statistical analysis

##### Condition comparisons

Condition-wise pupil time courses were obtained by averaging baseline-corrected z-scores across trials within each condition, followed by computing the across-participant mean and standard error. Time-course plots show the mean pupil z-score aligned to stimulus onset, with shaded error bands representing the standard error across participants (Figures 2 and 4, top panels). For scalar summaries, we defined a post-stimulus window of 0.5–2.0 s and averaged the pupil trace within this interval per trial.

##### Analysis of Variance (ANOVA)

For the standard analysis, trial-level pupil data were averaged within each condition for each participant to obtain subject-level means. In Experiment 1, both frequent and Bayesian three-way within-subject ANOVAs were conducted with the factors of Visibility (CFS vs. Visible), Gabor luminance (black vs. white), and Attended feature (luminance vs. orientation). In Experiment 2, attended Gabor luminance was defined based on the location of the RSVP stream, and the frequent and Bayesian two-way within-subject ANOVAs were conducted with the factors of Visibility (CFS vs. Visible) and Attended Gabor luminance (black vs. white). Bar plots summarize the 0.5–2.0 s window with corresponding standard errors (Figures 2 and 4, bottom panels). RSVP task performance was also summarized by computing the proportion of correct counts of the target letter in each condition.

##### Linear Mixed-Effects (LME) analysis

To robustly account for the unbalanced design (unequal number of trials between Visible and CFS conditions) and to control for potential oculomotor confounds, we conducted trial-level Linear Mixed-Effects (LME) models using the lme4 and lmerTest packages in R.

In Experiment 1, the LME model included Visibility, Gabor Luminance, and Attended Feature, along with their interactions, as fixed effects. In Experiment 2, the LME model included Visibility and Attended Gabor Luminance, along with their interaction, as fixed effects. For both experiments, we modeled random intercepts for each subject to account for repeated measures. Significance of fixed effects was assessed using Type III Analysis of Variance with Satterthwaite's method for degrees of freedom.

Crucially, to control for fixation stability and potential pupil-foreshortening errors (PFE),^32^ we included trial-by-trial horizontal (x) and vertical (y) gaze coordinates as continuous covariates in the models. We performed Likelihood Ratio Tests (LRT) to verify whether the inclusion of these gaze covariates significantly improved model fit compared to a reduced model excluding them.

##### Bayes Factor analysis

To quantify the strength of evidence for the null hypothesis (i.e., absence of attentional modulation under suppression), we calculated Bayes Factors (*BF*_01_) using data exclusively from the CFS condition. In Experiment 1, we compared an alternative model (*H1*) that included the Luminance × Attention interaction against a null model (*H0*) that included only the additive effects of Luminance and Attention (assuming no modulation). In Experiment 2, we compared an alternative model (*H1*) that included Attended Gabor Luminance against a null model (*H0*) that excluded this factor. A *BF*_01_> 3 was considered moderate evidence in favor of the null hypothesis.

##### Trial-level gaze-pupil correlation analysis (experiment 2)

While participants generally maintained central fixation, inspection of gaze density distributions (Figure S1) revealed a horizontal bias toward the task-relevant RSVP streams. Given this deviation, it was critical to determine whether the observed pupillary responses were triggered by spatial attention or merely by physical changes in retinal illuminance due to fixation instability. To address this, we performed a functional analysis of the relationship between gaze position and pupil size. For each trial, we calculated the Gaze-to-Stimulus Distance, which is the absolute horizontal gaze deviation relative to the center of the attended Gabor patch (|*Gaze*_*x*_-*TargetCenter*|). This metric quantifies the retinal eccentricity of the attended stimulus; smaller values indicate that the fovea is closer to the luminance source.

We then analyzed the linear relationship between this distance and pupil size separately for the Attend-Black and Attend-White conditions (Figure S2). We reasoned that if the PLR were driven solely by physical retinal illuminance, the functional relationships should be directionally opposite: closing the distance to the White Gabor (increasing light intensity) should cause constriction, whereas closing the distance to the Black Gabor (decreasing light intensity) should cause dilation. We inspected these relationships separately for the CFS and Visible conditions.
