# Supplementary material for: Pupillary responses to invisible brightness escape from attentional modulation
Source: iScience. 2026 Apr 28;29(6):115919. doi: 10.1016/j.isci.2026.115919 (PMC13207345; doi:10.1016/j.isci.2026.115919)
Supplement: Document S1. Figures S1 and S2 [file mmc1.pdf]

iScience, Volume *29*

Supplemental information

Pupillary responses to invisible brightness escape from attentional modulation

Yung-Hao Yang and Hsin-I Liao

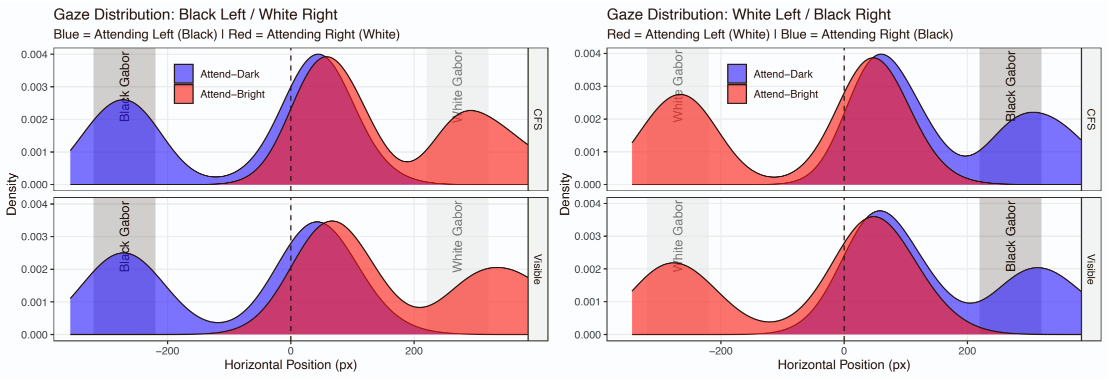

Supplementary Figure S1. Gaze density distributions during the spatial attention task (Experiment 2). Probability density plots represent horizontal gaze positions relative to the screen center (0°) for the Attend-Black (Blue) and Attend-White (Red) conditions. Data are faceted by Visibility (top: CFS; bottom: Visible) and Stimulus Configuration (left: black Gabor left/white Gabor right; right: white Gabor left/black Gabor right). While participants generally maintained central fixation, a horizontal bias toward the task-relevant RSVP streams is observed in both conditions. This deviation motivated the trial-level functional analysis (Figure S2) to support that the observed pupillary differences were not simply driven by physical changes in retinal illuminance. Related to STAR methods and the Experiment 2 Results session.

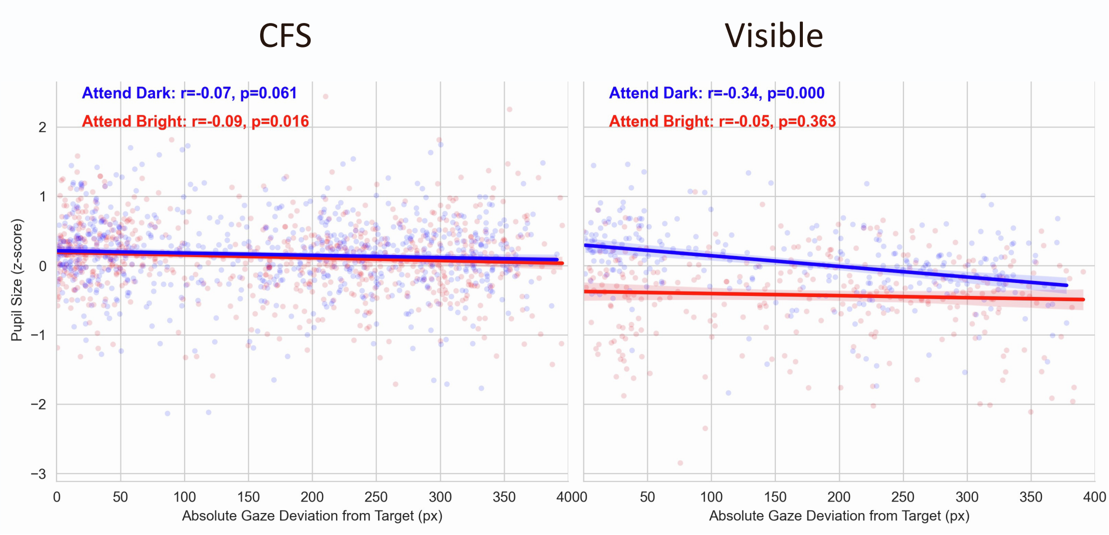

Supplementary Figure S2. Control analysis of gaze stability in Experiment 2. Scatter plots illustrating the relationship between absolute gaze deviation from the target (in pixels) and normalized pupil size (z-score) for the CFS (left panel) and Visible (right panel) conditions. Data are separated by the attended stimulus: Attend Dark (blue) and Attend Bright (red). Pearson correlation coefficients (*r*) and corresponding *p*-values are reported for each linear regression. Related to STAR methods and the Experiment 2 Results session.
